# Upper respiratory infections in a rural area with reduced malaria transmission in Senegal: a pathogens community study

**DOI:** 10.1186/s12879-018-3362-8

**Published:** 2018-09-10

**Authors:** Roger C. Tine, Léon A. Ndiaye, Mbayame N. Niang, Davy E. Kiori, Ndongo Dia, Oumar Gaye, Hélène Broutin

**Affiliations:** 1 Service de Parasitologie, Faculté de Médecine, Université Cheikh Anta Diop de Dakar (UCAD), BP5005 Dakar-Fann, Senegal; 2 Laboratoire des Virus respiratoires, Institut Pasteur de Dakar, Dakar, Senegal; 3 MIVEGEC, UMR CNRS -IRD -University of Montpellier - 911, Avenue Agropolis BP 64501, Cédex 5 34394 Montpellier, France

**Keywords:** ARI, Senegal, Pathogen community, *Streptococcus pneumoniae*, RSV, Influenza

## Abstract

**Background:**

Acute Respiratory Infections (ARI) are common causes of febrile illnesses in many settings in Senegal. These infections are usually managed presumptively due to lack of appropriate diagnostic tools. This situation, can lead to poor management of febrile illness or antibiotic misuse. In addition, there are limited data on the spectrum of pathogens commonly responsible for these ARI. This study was conducted to explore the pathogens community among patients with acute respiratory infection in a rural area in Senegal.

**Methods:**

A cross sectional study was conducted from August to December 2015. Children and adult patients attending Keur Socé health post for signs suggestive of acute respiratory infection were enrolled after providing inform consent. Eligible participants were recruited using a consecutive sampling method. Paired nose and throat swabs were collected for pathogen detection. Samples were processed using a multiplex PCR designed to identify 21 pathogens including both virus and bacteria.

**Results:**

Two hundred and fifty patients participated in the study. Samples positivity rate was evaluated at 95.2% (238/250). *Streptococcus pneumoniae* was the predominant pathogen (74%) and was present in all months and all age-groups, followed by *Staphylococcus aureus* (28,8%) and rhinovirus (28,4%). Respiratory syncytial virus (RSV) was detected only among children under 5 years old in August and September while coronavirus was present in all age groups, during the months of October and December.

**Conclusion:**

This pilot study revealed a diversity of pathogens over the time and across all age groups, highlighting the need for further exploration. A pathogen community approach including both virus and bacteria at a larger scale becomes crucial for a better understanding of transmission dynamics at population level in order to help shape ARI control strategies.

## Background

Progress in the fight against malaria achieved through increased deployment of insecticide-treated bed-nets and artemisinin combination therapy (ACTs) has led to significant reduction of the disease burden in many malaria-endemic countries [1–3]. In Senegal, malaria has been a major disease for many years, accounting for 35% of all outpatients consultations in health care services [4]. The introduction of rapid diagnostic tests (RDT) in the country, has contributed to a better documentation of malaria cases and has resulted in a reduction of antimalarial consumption [5]. While RDTs can improve targeting of anti-malarial drugs, there is no comparable test for bacterial or viral diseases and these pathogens are known to be leading causes of febrile illnesses [6]. Thus, patients with negative malaria RDT are being managed presumptively in many settings [7] and many febrile patients may be prescribed broad spectrum of antibiotics. In the context of declining of malaria reported in many African countries [8], this situation can lead to the occurrence and the spread of antibiotic resistance as well as poor management of febrile illnesses [9]. Thus, appropriate treatment guidelines are needed [10]. To achieve this, a better understanding in the epidemiology of non-malaria febrile illnesses is required. A prospective study conducted by the department of medical parasitology in Keur Socé rural area, a malaria sentinel site in the central part of Senegal, revealed that 75% of febrile illnesses among outpatients with negative malaria RDT were caused by acute respiratory infections (Tine et al. unpublished). However, the study was mainly a syndromic-based disease surveillance approach with limited biological investigations for pathogens identification. A better understanding of pathogens distribution and frequency during acute respiratory tract infections will improve current treatment guidelines and help shape preventive strategies related to these diseases.

Studies conducted in Africa, identified respiratory tract infections due to *Streptococcus pneumoniae* as a main cause of non-malaria febrile illnesses among other causes such as typhoid fever, urinary tract infection and other parasitic infections other than malaria [11–13].

Although several studies showed the importance of Acute Respiratory Infections (ARI) on febrile illness occurrence [11–13], few of them were conducted among adult patients [14, 15]. However, understanding ARI transmission dynamic may require additional investigations among higher age group population. Indeed the route transmission of these ARI is still unclear and looking at pathogens distribution across all age-groups has become crucial for a better understanding of pathogens reservoir, especially in rural settings with high burden of ARI such as the central part of Senegal. In the meantime, most of the previous studies focused on bacterial ARI and did not include assessment of viral causes like influenza, which is known to be present in all age-groups [16–18].

To provide first insights on these important gaps, we conducted a pilot study in Senegal, to explore the pathogens community in the nasopharyngeal tract among individuals with ARI in a rural primary health care unit, including all age groups and targeting both bacteria and viruses.

## Methods

### Study settings

The study was carried out at the rural area of Keur Socé, a malaria sentinel site located in the central part of Senegal, at 200 km from the capital city of Dakar. The area of “Keur Socé” covers 73 villages with 2 health posts providing mainly primary care (Keur Socé health post and Lamarame health post), headed by nurses. A demographic surveillance system (DSS) is going on within the 73 villages with a total population size at 35000 inhabitants.

### Study design and participants

A cross sectional study was carried out from August to December 2015. Children and adults attending the Keur Socé Health post for symptoms suggestive of acute respiratory infection according to IMCI guideline [10] were screened by the health post staff for eligibility. Eligible participants were then sent to the research team for enrolment in the study if they consented to participate. Participants, who were previously screened for respiratory tract infection within the same study period and patients with severe illness, were excluded. Eligible participants were recruited using a consecutive sampling method.

### Data collection methods

A code was given to each enrolled participant and a physician examined each participant prior to the biological assessments, which included naso-pharyngeal swab collection for PCR analysis. The patient was interviewed directly to determine socio-demographic characteristics, patient’s medical history and vaccination status, using a standard questionnaire. For children, these information were obtained from their mothers or care takers. For each enrolled study participant, paired nose and throat swabs were collected for pathogens detection. Symptoms presented by each participant on the day of survey, as well as data obtained from interviews and biological investigations were assigned on a case report form (CRF).

### Laboratory methods

Samples were collected by the study physician using a sterile nylon flocked swab and placed in viral transport medium *(“BD Universal Viral”),* labelled and transported on ice at Pasteur Institute Dakar for further processing and storage of the samples. Nucleic acids were extracted using the Qiamp viral Kit according to manufacturer’s instructions. Pathogens detection was performed using the Fast track Diagnostic Kit (FTD Respiratory pathogens 21 kit Luxembourg) which is a multiplex PCR essay designed to detect 21 pathogens. The kit presents 6 tubes multiplex PCR for the detection of different pathogens: (i) First tube: influenza A, influenza B, influenza A H1N1, human rhinovirus; (ii) second tube: coronavirus NL63, coronavirus 229E, coronavirus OC43, coronavirus (HKU1); (iii) third tube: parainfluenza 2, parainfluenza 3, parainfluenza 4, Internal Control; [19] fourth tube: parainfluenza 1, human metapneumovirus A/B, bocavirus, *Mycoplasma pneumoniae*; (v) fifth tube (respiratory syncytial viruses A/B, adenovirus, enterovirus, parechovirus; (vi) sixth tube: *Chlamydia pneumoniae*, *Streptococcus pneumoniae*, *Haemophilus influenzae* type B, *Staphylococcus aureus*. The multiplex real-time PCR FTD assay was performed on an ABI 7500 Fast instrument (Life Technologies, USA) as described elsewere [20] and PCR programme was as follow: 50 °C for 15 min, 95 °C for 10 min, 40 cycles of 95 °C for 8 s, 60 °C for 34 s.

### Statistical methods

Sample size assumption: with 250 participants sampled, the study was powered at 80% to provide an estimate for each pathogen with a 10% precision, assuming a 75% morbidity related to acute respiratory infection among febrile outpatients (temperature > 37 °C) seen at the health post (Tine et al. unpublished) with alpha at 0.05 (two sided).

Data were entered in Excel software and all analyses were conducted using Stata package (StataCorp, Texas). For categorical data, percentage was used to assess the frequency of each outcome with a 95% confidence interval. For continuous data, mean and standard deviation were used to describe normally distributed variables, median and range for other data. Characteristics of all participants included in the study were tabulated. Proportions were compared using chi square test or Fisher exact test where appropriated (univariate analysis). To assess the effect of age on pathogens distribution, a multivariate logistic regression was done with adjustment on covariates such as study period. From the final model, adjusted odds ratios were derived with their 95% confidence interval. Model validity was tested using the Hosmer-Lemeshow goodness of fit test. The performance of the final model was assessed by the area under the curve and Akaike and Bayesian information criterion; in addition a test for multicolinearity between variables was done using the variance inflation factor. Significance level of the different tests was 0.05, two sided.

### Ethical considerations

Participation to the study was strictly voluntary and patients who refused to be enrolled were not included in the study. A signed informed consent was obtained from each eligible participant prior to any investigation. For minor participants (age < 18 years) informed consent was obtained from parents or children’s legal representative prior to their enrollement. To ensure confidentiality, information collected during the study were analysed using participant’s identification code. The study protocol and other study related documents (CRF, Inform consent Form) were approved by the Senegalese national ethics committee (*Conseil National d’Ethique et de Recherche en Santé* – *N°0166 MSAS/DPRS/CNERS du 10 Mai 2014)*.

## Results

### Participant’s characteristics at enrollment

Among the 565 patients who consulted at the health post for signs suggestive of respiratory infections, 250 of them fulfilled all defined inclusion criteria and were enrolled in the study. The majority of the samples were collected among the 1 to 5 years age-group which also represents the most affected age-group. Due to a very low number of samples in the 6–10 years (8 samples) and 11–15 years (6 samples), we aggregated both age groups in an unique age-group (6–15 years) for subsequent analyses (Table 1).

**Table 1:** Number of samples done by month and by age group from August to December 2015

Month	< 1 year	1–5 years	6–15 years	> 15 years	Total
August	15	29	1	5	50
September	11	28	4	7	50
October	12	23	4	11	50
November	16	20	1	13	50
December	17	23	4	6	50
Screened at health post for eligibility	160	223	49	130	565
Enrolled in the study	71	123	14	42	250

### Frequency and spectrum of identified pathogens

**Fig. 1:**
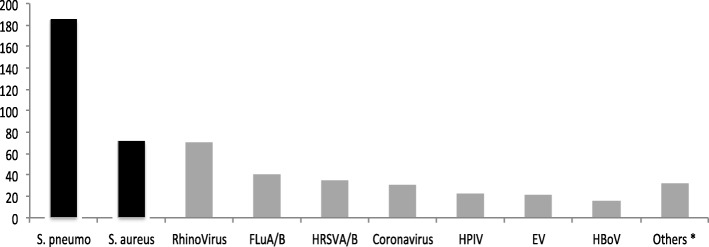
Pathogens distribution among the 250 analysed samples

Among the 250 samples, at least one pathogen was identified in 238 samples (95,2%) and the most frequent pathogen identified was S*treptococcus pneumoniae* (185/250, 74%), followed by *Staphylococcus aureus* (72/250, 28,8%) and *rhinovirus* (71/250, 28,4%) (Fig. 1).

Overall, 124 participants (49.6%) were found with one viral species while 44 patients (17.6%) were infected by at least 2 viruses; a proportion of 2.8% (7 patients) were co-infected with 3 viruses. Patients with one bacterial species represented 55.6% while that was at 27.2% for patients with 2 bacterial species. In total, 238 patients were found with at least 1 bacteria and 1 virus at the same time (Table 2).

**Table 2:** Overall distribution of sample positivity and pathogens association (*N* = 250)

Type of pathogenes	Number	Percentage	(95%CI)
Virus			
Absence	75	30	24.4–36.1
patients with viral species	124	49.6	43.2–55.9
patients carrying 2 viral species	44	17.6	13.1–22.9
patients with 3 viral species	07	02.8	1.1–5.7
Combined (at least 1 viral pathogen)	175	70	63.9–75.6
Bacteria			
Absence	43	17.2	12.7–22.5
patients with 1 bacterial species	139	55.6	49.2–61.8
Patients with 2 bacterial species	68	27.2	21.8–33.2
Combined (at least 1 bacterial pathogen)^a^	207	82.8	77.5–87.3
Association of virus and bacteria			
Absence	12	4.8	2.5–8.2
Patients with at least 1 virus and 1 bacterial species	238	95.2	91.8–97.5

^a^Comparison between proportion of patients with at least one viral pathogen versus patients with bacterial pathogen: difference = 12.8% (95%CI (5.4–20.2) *p* = 0.001)

The highest number of pathogens was detected among the under 5 years age group; these pathogens were mainly represented by *Streptococcus pneumoniae*, rhinovirus (in < 1 year) and *Staphylococcus aureus* (1-5 years). Among patients with age above 15 years, the main pathogens detected at the nasopharyngeal level were rhinovirus (31%) and *Staphylococcus aureus* (28,6%). *Streptococcus pneumoniae* was found in all age groups, with a higher frequency among infants and children (i.e. > 70%); it was also identified among patients above the age of 15 years with a frequency of 23.8%. Similarly, rhinovirus was detected in all age groups with lower proportion among the above 15 years old age-group (13/71, 18%). These 2 pathogens are thus circulating in all age groups. In contrast, human respiratory syncytial virus was detected only among the less than 5 years old age-group. The majority of influenza A/B (27/40, 67,5%) and coronavirus (14/31, 45.1%) were detected in 1–5 years old age group. The rest of the pathogens were detected at low levels in all age-groups. The distribution of the identified pathogens by age group is described in Table 3.

**Table 3:** Spectrum of the identified pathogens from naso-pharyngeal swabs by age-groups

Age-group	*S. pneumo* *(n = 185)*	*S. aureus* *(n = 72)*	*RhinoVirus* *(n = 71)*	*FLu* *A/B* *(n = 40)*	*HRSV* *A/B* *(n = 35)*	*Corona-virus* *(n = 31)*	*HPIV* *(n = 22)*	*EV* *(n = 21)*	*HBoV* *(n = 16)*	Others^a^ *(n = 32)*	*Total number of pathogens identified*
< 1 year (n = 71)	**61 (** ***85,9*** **)**	16 (*22,5*)	**29 (** ***40,8*** **)**	6 (*8,5)*	14 (*19,7*)	10 (*14,1*)	7 (*9,9*)	5 (*7*)	4 (*5,6*)	9 (*12,7*)	***161***
1–5 years (*n* = 123)	**104 (** ***84,6*** **)**	**37 (** ***30,1*** **)**	27 (*22,0)*	27 (*22,0)*	20 (*16,3)*	14 (*11,4*)	11 (*8,9*)	8 (*6,5*)	6 (*4,9*)	16 (*13,0*)	***270***
6–15 years (*n* = 14)	**10 (** ***71,4*** **)**	**7 (** ***50,0*** **)**	2 (*14,3*)	4 (*28,6*)	0	2 (*14,3*)	2 (*14,3*)	3 (*21,4)*	2 (*14,3*)	2 (*14,3*)	***34***
> 15 years (*n* = 42)	10 (*23,8*)	**12 (** ***28,6*** **)**	**13 (** ***31,0)***	3 (*7,1*)	1 (*2,4*)	5 (*11,9)*	2 (*4,8*)	5 (*11,9)*	4 (*9,5*)	5 (*11,9*)	***60***

*The number in italic corresponds to percentage (%) among the age-groups. Bold numbers represent the 2 main pathogens identified in the considered age-group. S. pneumo*: *Streptococcus pneumoniae; H. Saureus: Staphylococcus aureus; Flu A/B: Influenza A/B; HRSV A/B: Human Respiratory Syncytial viruses, HPIV Human ParaInfluenza Virus, EV Enterovirus, BoV Bocavirus*

^*a*^ *Others: Human metapneumovirus (HMPV) A/B, H1N1, Mycoplasma pneumoniae, Human Parechovirus (HPeV), Human Adenovirus (HAdv), Chlamydia pneumoniae, Haemophilus influenzae type B*

### Pathogens distribution by age group and period

**Fig. 2:**
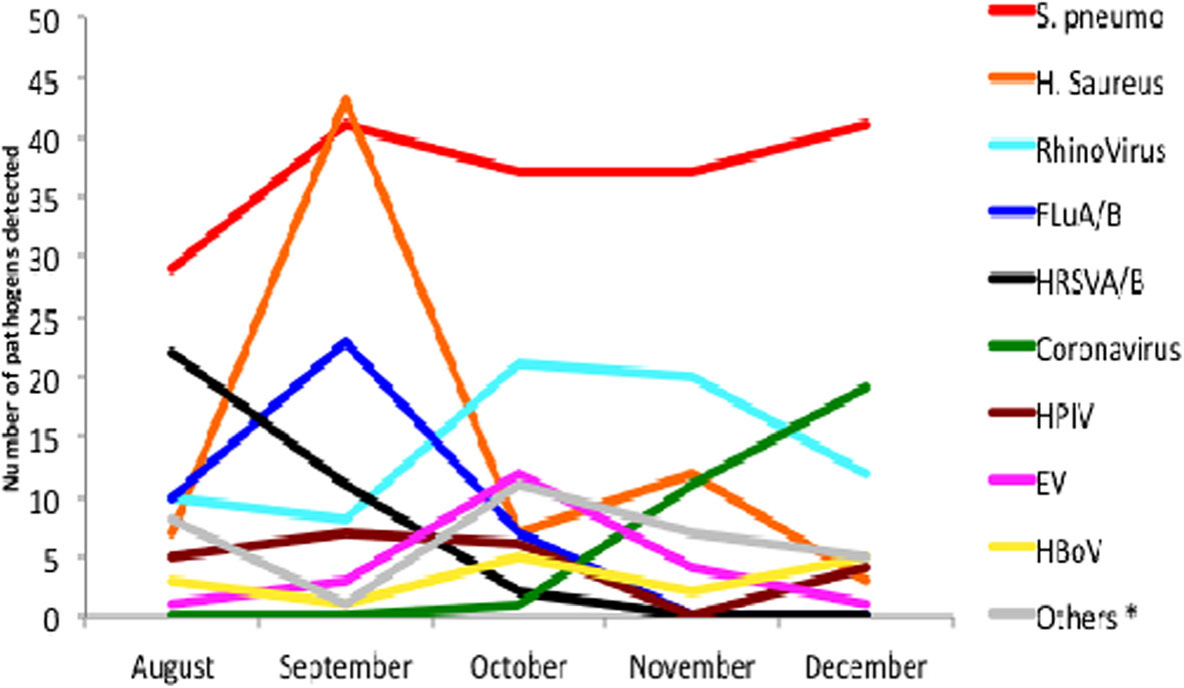
Monthly Distribution of pathogens (2015)

The 5 months sampling provided new insights about the temporal dynamics of pathogens. *Streptococcus pneumoniae* was detected in similar proportion during the 5 months and remained the major pathogen detected (Fig. 2). The majority of human respiratory syncytial virus (22/35, 63%) was detected in august, then it decreased in september and October; no respiratory syncytial virus was found during the months of november and december. *Staphylococcus aureus* and influenza virus A/B increased in september then decreased during the rest of the year. Rhinoviruses were detected at a low level in august and September; its frequency increased in october and november and then decreased in december. Finally, coronavirus were only detected from october and increased continuously up to december. All this temporal distribution shows a trend of successive pathogens over time within the population.

Overall, bacterial pathogens were more common among children compared to viral pathogens with significant statistical differences among children less than 1 year (prevalence difference: 34.1%; *p* = 0.001), and 1 to 5 years (prevalence difference: 19.5%; *p* = 0.0002); no significant difference was noted when comparing the frequency of bacterial versus viral pathogens both in the 6 to 15 years (prevalence difference: 28.8%; *p* = 0.06) and in the > 15 years (prevalence difference: 9.5%; *p* = 0.38) (Table 4).

**Table 4:** Pathogens distribution by age group and study period

	Viral pathogenes			Bacterial pathogenes			p value
	Number examined	Positive	Prevalence (95%CI)	Positive	Prevalence (95%CI)	Prevalence difference (95%CI)	
Age categories							
< 1 year	71	56	78.9 (67.5–87.7)	64	90.1 (80.7–95.9)	34.1 (20.6–47.6)	0.0001
1–5 years	123	85	69.1 (60.1–77.1)	109	88.6 (81.6 - 93.6)	19.5 (9.6–29.4)	0.0002
6–15 years	14	09	64.2 (35.1–87.2)	13	92.8 (66.1–99.8)	28.6 (0.06–57.1)	0.06
> 15 years	42	25	59.5 (43.3–74.4)	21	50.0 (34.2–65.8)	9.5 (11.7–30.7)	0.38
Study period							
August	50	44	88.0 (75.7–95.5)	33	66.0 (51.2–78.8)	22 (6.1–39.9)	0.009
September	50	29	58.0 (43.2–71.8)	49	98 (89.3–99.9)	40 (25.8–54.2)	< 0.001
October	50	41	82.0 (68.6–91.4)	40	80 (66.3–89.9)	02 (13.4–17.4)	0.79
November	50	30	60.0 (45.2–73.6)	42	84 (70.9–92.8)	24 (7.1–40.9)	0.007
December	50	31	62.0 (47.2–75.3)	43	86 (73.2–94.2)	24 (7.5–40.5)	0.006

A higher frequency of viral pathogens compared to bacterial pathogens was noted during the month of august (prevalence difference: 22%; *p* = 0.009). During the months of september, november, and december, bacterial pathogens were predominantly isolated compared to virus (all *p values* for these periods are < 0.05) (Table 5). Adjusted on age group, patients were less likely to carry viral infections during the months of september (adjusted Odds ratio:0.19, 95%CI(0.07–0.55)), november (adjusted Odds ratio: 0.22, 95%CI(0.08 - 0.64)) and december (adjusted Odds ratio: 0.22, 95%CI(0.08–0.63)) (Table 5).

**Table 5:** Adjusted effect of age and period on pathogens carriage among the study participants

Presence of virus				
	Frequency (95%CI)	OR (95%CI)	aOR (95%CI)	*p* value
Age categories				
< 1 year	78.9 (67.5–87.7)	*Reference*	*Reference*	
1–5 years	69.1 (60.1–77.1)	0.60 (0.30–1.19)	0.58 (0.28–1.19)	0.13
6–15 years	64.2 (35.1–87.2)	0.48 (0.14–1.65)	0.52 (0.14–1.87)	0.32
> 15 years	59.5 (43.3–74.4)	0.39 (0.17–0.97)	0.39 (0.16–0.94)	0.04
Study period				
August	88.0 (75.7–95.5)	*Reference*	*Reference*	
September	58.0 (43.2–71.8)	0.19 (0.07–0.52)	0.19 (0.07–0.55)	0.002
October	82.0 (68.6–91.4)	0.56 (0.19–1.68)	0.61 (0.20–1.85)	0.38
November	60.0 (45.2–73.6)	0.21 (0.08–0.60)	0.22 (0.08–0.64)	0.005
December	62.0 (47.2–75.3)	0.22 (0.08–0.62)	0.22 (0.08–0.63)	0.005
Presence of bacteria				
	Frequency (95%CI)	OR (95%CI)	aOR (95%CI)	*p* value
Age categories				
< 1 year	90.1 (80.7–95.9)	*Reference*	*Reference*	–
1–5 years	88.6 (81.6 - 93.6)	0.85 (0.33–2.22)	0.82 (0.30–2.24)	0.69
6–15 years	92.8 (66.1–99.8)	1.42 (0.16–12.56)	0.95 (0.09–9.25)	0.97
> 15 years	50.0 (34.2–65.8)	0.11 (0.04–0.29)	0.05 (0.02–0.18)	< 0.001
Study period				
August	66.0 (51.2–78.8)	*Reference*	*Reference*	–
September	98 (89.3–99.9)	25.2 (3.5–45.8)	56.5 (6.19–60.8)	< 0.001
October	80 (66.3–89.9)	1.87 (0.77–4.55)	3.55 (1.21–10.39)	0.02
November	84 (70.9–92.8)	3.09 (1.15–8.33)	8.66 (2.47–30.44)	0.001
December	86 (73.2–94.2)	3.16 (1.17–8.52)	4.51 (1.45–14.02)	0.009

*Goodness of fit: chi (8df) = 3.74; p = 0.88. Area under the curve: 83.4%. Akaike information criterion (AIC) = 298.26; Bayesian information criterion = 326.43. Variance inflation factor (VIF) = 1.48*

## Discussion

Acute Respiratory Infections are usually a clinical diagnosis in many primary health care units in Senegal. The diagnosis approach is often based on IMCI guidelines [10]. This pilot study was conducted to assess the spectrum and frequency of pathogens responsible of ARI using a highly sensitive multiplex RT-PCR technique. The study revealed a high positivity rate both for viral and bacterial pathogens and a broad spectrum of pathogens were detected in all age groups over the 5 months period of data collection, providing an original pathogens community study in the global population.

*Streptococcus pneumoniae* was the main pathogen detected and it remained constantly highly present over the 5 months of study, mainly among children but also among adults, suggesting a non-negligible and permanent reservoir of this pathogen among older age-groups. This observation deserves further investigation since it suggests that adults represent an important fraction of S*treptococcus pneumoniae* reservoir. The potential contribution of these adults in the transmission of *Streptococcus pneumoniae* to younger children needs to be investigated to determine whether they also need protection or not. Unfortunately, the study could not assess the diversity of the different pneumococcal serotypes in order to determine serotype variability across age-groups. In the context of recent introduction of pneumococcal conjugated vaccine-13 (PCV-13) in the Senegalese immunization program, it is now crucial to document the circulating serotypes, compared to the vaccine-serotypes, in all age-groups. This will help to generate evidence on the impact of vaccination on pneumococcal serotypes.

The highest diversity of pathogens detected from nasopharyngeal swab among patients with signs suggestive of ARI was observed among children under 5 years old. Indeed, under five children remained the most vulnerable group with regard to ARI as describe in other studies [14, 21]. *Streptococcus pneumoniae*, *Staphylococcus aureus* and *rhinovirus* were the major pathogens detected in all age-groups. In contrast, *respiratory syncytial virus* has been detected only in < 5 years old (except in 1 adult) children, suggesting a transmission route of the pathogen among this specific population group.

The 5 months study has also suggested a temporal dynamics of the pathogens with different seasonal patterns. For instance *respiratory syncytial virus* was only observed in august and september, while coronavirus was detected from october to december. That temporal dynamic of pathogens needs to be further investigated throughout a continuous surveillance system over several years, to look at potential seasonality of these pathogens. Establishing a clear temporal dynamic of pathogen could help shape treatment practices and preventive interventions.

This study presents some caveats. The first limit consists in the length of the study, which is not large enough to clearly establish a seasonally. Nevertheless, this pilot study revealed relevant trends to explore further, considering the specific pattern of pathogens (e.g. human respiratory syncytial virus, coronavirus) or the age-groups distribution of pathogens (e.g. *Streptococcus pneumoniae* vs human respiratory syncytial virus) to better understand the transmission dynamics.

The study used a highly sensitive multiplex RT-PCR to detect pathogens among patients with signs suggestive of ARI. However, it was not possible to clearly establish which of the identified pathogen was responsible of the observed symptoms, but still all these pathogens are circulating within the whole population.

*Streptococcus pneumoniae* remains the major pathogen circulating in all age groups over the 5 months of study. The next step consists in determining the serotypes that are circulating and responsible for ARI in the context of the recent introduction of the Pneumococcal vaccines in Africa, in the immunization programs. Only infants receive vaccines and the seroconversion is quick. It is perfectly timely to follow on the evolution of the pneumococcal serotypes under vaccine pressure in the whole population. *Streptococcus pneumoniae* carriage among adults is not negligible in our study highlighting the need for further investigations on adult’s role in *Streptococcus pneumoniae* transmission to infants.

## Conclusion

This first pilot study of 5 months surveillance at the community level, including both virus and bacteria deserves to be routinely implemented to assess the spatial and temporal dynamics of the pathogens community at the nasopharyngeal level. Such a surveillance system will allow improvement of current treatment practices and may contribute to reduce antibiotic consumption. Public health programs need a community pathogens overview to better control respiratory infections among high-risk groups. Co-infections are crucial to explore further to better understand the link between symptoms and pathogens and be able to provide appropriate treatment guidelines.
